# Editorial: Archaeal Cell Envelope and Surface Structures

**DOI:** 10.3389/fmicb.2015.01515

**Published:** 2016-01-06

**Authors:** Mechthild Pohlschroder, Sonja-Verena Albers

**Affiliations:** ^1^Department of Biology, University of PennsylvaniaPhiladelphia, PA, USA; ^2^Department of Microbiology, Institute of Biology, University of FreiburgFreiburg, Germany

**Keywords:** archaea, membrane, S-layer, surface filaments

Archaea and Bacteria have complex cell envelopes that play important roles in several vital cellular processes, including serving as a barrier that protects the cytoplasm from the environment. Along with associated proteinaceous structures, cell envelopes also ensure cell stability, promote motility, mediate adherence to biotic and abiotic surfaces, and facilitate communication with the extracellular environment. While some aspects of the biosynthesis and structure of the cell envelope are similar across the three domains of life, archaeal cell envelopes exhibit several unique characteristics. Moreover, recent analyses have revealed that many features of cell envelopes can vary greatly between distantly related archaea. The collection of reviews and original research papers in this focused issue describes research that has significantly expanded our understanding of the mechanisms underlying the biogenesis and functions of archaeal cell envelopes and their constituent surface structures.

Jain et al. provide a comprehensive review of our current knowledge of the unique archaeal cytoplasmic membrane, an isoprenoid lipid bilayer, as well as recently revealed aspects of cytoplasmic membrane biosynthesis that are conserved across the three domains of life. Complementing this review, Andreas Klingl summarizes the diverse structures and functions of archaeal cytoplasmic membranes (Klingl). While most archaeal cells have a single membrane, archaea having an outer membrane, which had been thought to be rare among archaea, have now been identified in a diverse variety of archaeal lineages. One particularly intriguing diderm is the hyperthermophilic archaeon *Ignicoccus hospitalis*, which has an outer cellular membrane that is energized and is able to use the electrochemical gradient across the membrane to synthesize ATP in the periplasmic space. Complementing this work, Kletzin provides an in-depth review of evolutionarily conserved and unique archaeal inner and outer membrane associated cytochromes (Kletzin et al.). The periplasmic space between the membranes of archaeal diderms does not contain a peptidoglycan layer. In fact, while the cytoplasmic membrane is superimposed by an S-layer in many monoderm archaea, it is unclear how diderms, and even some monoderm extremophiles that lack an S-layer, withstand osmotic stress. As noted by Klingl, glycocalyx, lipoglycans, or other protective cell-associated glycoproteins, may take on the functions of a cell wall in some archaea. One such secreted protein, as described by Zenke et al., is the halomucin of *Haloquadratum walsbyi* (Zenke et al.). While *H. walsbyi* does have a cell wall, halomucin, an unusually large protein (9159 aa), is thought to play an important role in protecting these extreme halophiles against desiccation.

Interestingly, *Candidatus Altiarchaeum hamiconexum*, an uncultured diderm euryarchaeon, isolated from biofilms contain hami, cell surface proteins with the appearance of grappling hooks that connect cells to each other and to abiotic surfaces. Perra's stunning imaging suggests that these hook-like filaments are connected to the inner membrane, and, surprisingly, are composed of subunits that share homology with S-layer glycoproteins, possibly suggesting a case of divergent evolution (Perras et al.).

Unlike hami, which appear to be limited to a subset of archaea, type IV pili, as pointed out by Pohlschroder and Esquivel as well as Losensky et al. are conserved across the prokaryotic domains, being found in the majority of sequenced archaea, where, as in bacteria, they play key roles in processes necessary for biofilm formation (Losensky et al.; Pohlschroder and Esquivel). Interestingly, as discussed by Albers and Jarrell, as well as Nather-Schindler et al., a type IV pilus-like structure is responsible for swimming motility in archaea.

Many secreted proteins, including the S-layer glycoprotein and pilin-like proteins, are heavily post-translationally modified. The known proteolytic modifications of the proteins of the model haloarchaeon *H. volcanii*, as reviewed here by Gimenez et al., highlight evolutionarily conserved characteristics, as well as well as the novel aspects, of these haloarchaeal proteases and their substrates. Using the results of proteomic studies, Leon et al. expand upon the existing experimental datasets of mature archaeal *N*-termini in the methanogen *Methanosarcina mazei* (Leon et al.), providing an invaluable resource for improving *in silico* prediction tools for the characterization of archaeal proteins, in general, but also specific phyla. Kandiba and Eichler review our current knowledge of N-glycosylation in archaea, including descriptions of the pathways the regulatory roles this post-translational modification plays in cellular processes (Kandiba and Eichler).

Considering the unique aspects of the archaeal cell envelope, including not only the protein structures, but their post-translational modifications as well, it is not surprising that archaeal viruses have evolved specific mechanisms to infect and egress from archaeal cells, which are reviewed in this issue by Quemin and Quax.

Understanding the roles that cell surfaces play in archaeal cellular processes can lead to important insights into the types of adaptations that allow some archaea to thrive in extreme environments, including the ability to form biofilms, which many archaea, including mucosa-associated methanogenic archaea, can establish, as described in this issue by Bang et al.. Archaeal cell membranes and S-layer glycoproteins have been used to make liposomes, and hami are also a potentially useful tool for nanobiological applications. Finally, a better understanding of the similarities and differences among the archaea as well as between the archaea and the other two domains will lead to the development of a more accurate phylogeny. In this issue, Forterre takes advantage of the recent profusion of genome studies, along with supporting *in vivo* work, to assemble an improved tree of life (Forterre).

## Author contributions

MP and S-VA have both written the text.

### Conflict of interest statement

The authors declare that the research was conducted in the absence of any commercial or financial relationships that could be construed as a potential conflict of interest.

